# Association between body fat composition and disease duration, clinical activity, and intravenous corticosteroid-induced response in inflammatory bowel disease

**DOI:** 10.1186/s12944-023-01874-4

**Published:** 2023-07-22

**Authors:** Shubei He, Yuxia Huang, Ying Peng, Jin Chai, Kun Chen

**Affiliations:** 1 Department of Gastroenterology, The First Affiliated Hospital (Southwest Hospital) to Third Military Medical University (Army Medical University), Chongqing, 400038 China; 2 Institute of Digestive Diseases of PLA, The First Affiliated Hospital (Southwest Hospital) to Third Military Medical University (Army Medical University), Chongqing, 400038 China; 3 Cholestatic Liver Diseases Center, The First Affiliated Hospital (Southwest Hospital) to Third Military Medical University (Army Medical University), Chongqing, 400038 China; 4 Center for Metabolic Associated Fatty Liver Disease, The First Affiliated Hospital (Southwest Hospital) to Third Military Medical University (Army Medical University), Chongqing, 400038 China

**Keywords:** Inflammatory bowel disease, Computed tomography, Body fat composition, Intravenous corticosteroids, Medical response, Imaging biomarkers

## Abstract

**Background:**

Body fat composition is believed to be associated with the progression, medical response, and prognosis of inflammatory bowel disease (IBD). Hence, we conducted this study to explore if fat metrics were associated with the disease activity of severe IBD and the response to intravenous corticosteroids (IVCS).

**Methods:**

We included 69 patients with ulcerative colitis (UC) and 72 patients with Crohn's disease (CD) who had previously received IVCS during hospitalization. We quantified individual fat distribution using abdominal computed tomography slices. The correlations between fat parameters and disease activity were available with Spearman correlation analysis. The prediction model was developed using independent risk factors derived from multivariable logistic regression analysis. Model discrimination was evaluated leveraging the receiver operating characteristic curve. 1000 bootstrap resamples internally validated the model's prediction performance.

**Results:**

Notable differences in age, nutritional status, serum cytomegalovirus replication, stool condition, and extraintestinal involvement between UC and CD patients were observed. UC subjects who responded to IVCS had higher subcutaneous adipose tissue index (SATI), visceral adipose tissue index (VATI), and mesorectal adipose tissue index (MATI) than non-responders. IVCS-responding CD individuals had lower VATI and mesenteric fat index (MFI) than non-responders. CD patients with a prolonged disease duration had a decreased SATI and an elevated MFI. VATI and MATI were reduced as UC clinically progressed, while more prominent clinical activity in CD correlated with increased VATI, MATI, and MFI. A high SATI indicated that patients with UC were more prone to be IVCS responders. For patients with CD, levels of VATI and MFI were negatively associated with effective IVCS treatment. The established models showed a discriminative accuracy of 0.834 [95% confidence interval (CI) 0.740–0.928] in the UC cohort and 0.871 (95% CI 0.793–0.949) in the CD cohort. Repeated samples supported the reliability of the developed models (AUC_UC_ = 0.836, 95% CI 0.735–0.919; AUC_CD_ = 0.876, 95% CI 0.785–0.946).

**Conclusion:**

Human fat indexes represent novel imaging biomarkers for identifying IBD patients who respond to IVCS, thus building accelerated therapy regimens and avoiding the adverse effects of ineffective IVCS.

**Supplementary Information:**

The online version contains supplementary material available at 10.1186/s12944-023-01874-4.

## Background

As a chronic idiopathic intestinal disorder, inflammatory bowel disease (IBD) comprises ulcerative colitis (UC) and Crohn's disease (CD), which clinically manifests as abdominal pain, diarrhea, fatigue, fever, and weight loss [1]. Due to its increasing global incidence rates, IBD poses a significant health burden [2]. Although IBD generally presents as a chronic and intermittent course, approximately one-quarter of patients will experience at least one acute severe episode [3], requiring hospital monitoring and intensive therapy. Intravenous corticosteroids (IVCS) have been acknowledged as the primary choice for managing severe UC and CD, although their efficacy in improving symptoms is suboptimal in some instances [4, 5]. In cases where short-term IVCS fail to achieve satisfactory remission, rescue treatments with ciclosporin and tumor necrosis factor antagonists or emergent surgeries are required [4, 5]. The timely and accurate identification of the steroid-refractory population is clinically helpful. A more rapid regimen of tumor necrosis factor antagonists has been shown to reduce the colectomy rate [6, 7], thereby avoiding colonic dysfunction and minimizing the threat of postoperative mortality [8]. The delayed surgical intervention leads to a higher risk of infectious and non-infectious postoperative complications [9, 10], which may also be triggered by an ineffective continuation of IVCS [11].

Computed tomography (CT) is an imaging-based supplementary examination that precisely quantifies body composition, especially skeletal muscle and fat. Recent studies have highlighted the potential of adipose tissue. As an endocrine organ, adipose tissue secretes multiple cytokines known as adipokines, which regulate inflammation, endocrine function, and metabolism [12]. Subcutaneous and visceral fat constitute the most important fat compartments of the human body, with their CT-derived morphological changes correlated with the disease progression, treatment response, and prognosis of IBD [13–16]. Other measures, such as paraspinal intramuscular and mesorectal fat areas, have been linked to various inflammatory diseases [17, 18]. Although crosstalks between adipokines and steroid-signaling pathways have been elucidated, fat measures are seldom utilized in evaluating IVCS-induced response.

Therefore, we aimed to investigate whether fat indicators could reliably identify IVCS-induced response in a cohort of acute severe IBD patients, thereby facilitating assessing the requirement for medical rescue therapies or surgeries. In addition, we examined the relationship between disease severity and adiposity parameters, bridging knowledge gaps between nutritional metabolism and IBD.

## Method

### Patient collection

**Fig. 1:**
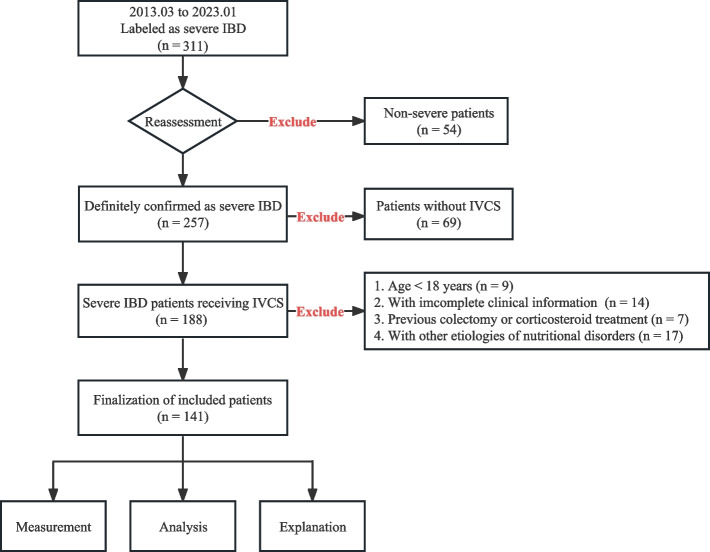
Research flowchart. IBD, inflammatory bowel disease; IVCS, intravenous corticosteroids

The study population consisted of patients with IBD who presented at the First Affiliated Hospital to Army Medical University between March 2013 and January 2023. The presence of acute severe UC was determined based on the Truelove and Witts criteria [19], which include more than six episodes of bloody diarrhea per day and at least one of the following: erythrocyte sedimentation rate greater than 30 mm/h, fever exceeding 37.8 °C, hemoglobin level less than 10.5 g/dL, or heart rate over 90 beats per minute. Patients with CD who presented significant malnutrition, persistent systemic toxicity, complicated phenotypes, or prominent intestinal and extraintestinal appearances were classified as severe [5, 20]. We extracted individuals with severe conditions who accepted intravenous methylprednisolone and hydrocortisone during hospitalization. Upon admission, participants underwent endoscopic examinations and abdominal CT scans. The following patients were excluded: 1) those who were under 18 years of age; 2) those with incomplete clinical information; 3) those who had undergone colonic resection or corticosteroids use; and 4) those with nutritional disorders as confounding factors, including chronic liver diseases, tuberculosis, and malignant tumors. Fig. 1 provides a detailed view of all available information acquired during the patient enrollment process.

### Adipose tissue measurement

**Fig. 2:**
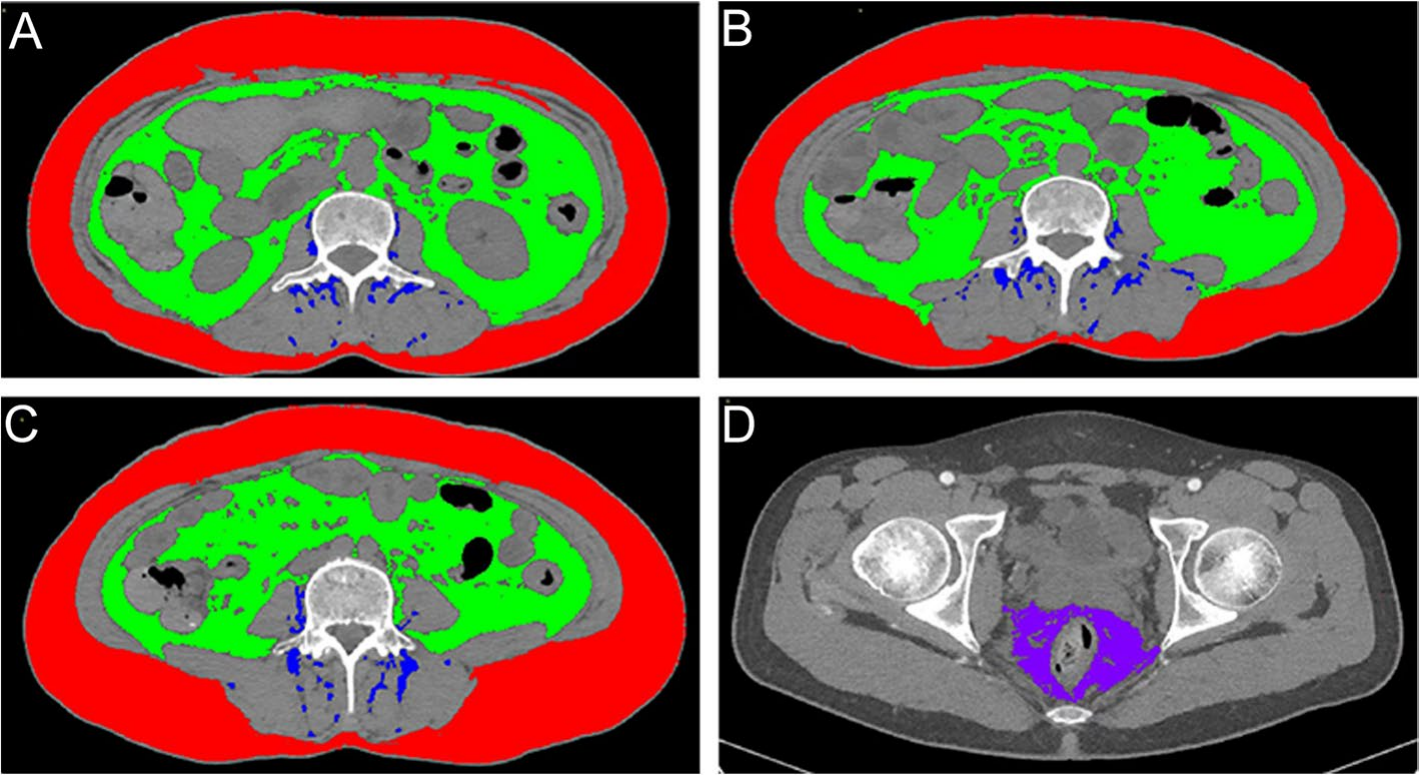
Diagram of the human fat compartments. **A**–**C**. Subcutaneous, visceral, and paraspinal intramuscular fat areas were measured at L2, L3, and L4. **D**. Mesorectal fat measured at the tip of the ischial spines. Subcutaneous fat is shown in red, while green and blue represent visceral and paraspinal intramuscular fat. L2, second lumbar vertebra; L3, third lumbar vertebra; L4, fourth lumbar vertebra

Measurement strategies should be tailored to accommodate the physiological and anatomical characteristics of different adipose compartments. A cross-sectional measurement of subcutaneous, visceral, or paraspinal intramuscular adipose tissue at the third lumbar vertebra accurately reflects whole-body fat mass [21, 22], which is less stable due to individual variations and errors in slice selection. Therefore, they were recorded at the second to fourth spinal levels and integrated. The tip of the ischial spines was chosen as a bony landmark to quantify mesorectal adipose tissue [23]. The SliceOmatic 5.0 software (Tomovision, Montréal, Canada) processed all scans and automatically computed the horizontal areas of adipose tissue compartments based on Hounsfield unit values. We traced the boundaries of adiposity regions and corrected the segmented bias in each slice. Adipose tissue zones are illustrated in Fig. 2. To standardize the estimated areas, they were normalized by dividing with the square of the height and expressed in cm^2^/m^2^, further producing subcutaneous adipose tissue index (SATI), visceral adipose tissue index (VATI), paraspinal intramuscular adipose tissue index (PSAI), and mesorectal adipose tissue index (MATI). We have calculated the mesenteric fat index (MFI), which denotes the ratio of visceral fat area and subcutaneous fat area [14].

The measurements were performed by two experienced physicians who were blinded to patients' clinical information and averaged (Fig. S1). CT images were acquired with a Siemens 64-slice SOMATOM Definition Flash dual-source machine and downloaded in dicom format from picture archiving and communication systems. The Hounsfield unit thresholds for SATI ranged from − 190 to − 30 [24], for VATI and MATI ranged from − 150 to − 50 [25], and for PSAI ranged from − 150 to − 30 [22].

### Demographic and clinical data

General materials on admission (age, sex, weight, height, temperature), baseline laboratory indexes [erythrocyte sedimentation rate, C-reactive protein, hemoglobin, albumin, cytomegalovirus (CMV) serology], and disease characteristics (duration, location, behavior, fecal condition, extraintestinal manifestation) were collected. We adopted the Simple Clinical Colitis Activity Index (SCCAI) [26] or Harvey-Bradshaw Index (HBI) [27] to score each patient, while mucosal inflammation was evaluated based on the UC Endoscopic Index of Severity (UCEIS) [28] or the Simple Endoscopic Score for CD (SES-CD) [29].

### Definition of the outcome event

The response to corticosteroids was assessed after 3–5 days (time point for UC) [20] and 7–10 days (time point for CD) with high-dose IVCS [30]. Patients were categorized as non-responders or responders based on if rescue therapies or surgeries were introduced before hospital discharge. Patients with non-regressed symptomatic and biochemical performance who quit further treatments were also defined as having inadequate clinical response.

### Statistical analysis

Intergroup comparison of baseline data and identification of independent risk factors were performed using SPSS 26.0 software (IBM Corp., Armonk, NY, USA). Spearman correlation analysis and model evaluation were accomplished in R Studio 1.4.1717 software (RStudio Inc., Boston, MA, USA). Continuous data with a normal distribution (tested by the Shapiro–Wilk method) were shown as means ± standard deviations, while non-normally distributed variables were described as medians with 25% and 75% quartiles. Their between-group variations were examined with the independent samples *t* test or Mann–Whitney *U* test. Percentages were used to represent categorical variables, which were analyzed by the Fisher exact test. We adopted Spearman correlation analysis to disclose the relationship between fat parameters and disease severity and considered a significant correlation if the *P*-value < 0.05.

The variance inflation factor (VIF) of variables to be analyzed in the regression equations was computed, and its value greater than 5 confirmed the presence of statistical multicollinearity [31]. Candidates with a *P*-value < 0.05, identified through univariable logistic regression, were included in a stepwise multivariable analysis to acquire robust risk factors of steroid-refractoriness in patients with severe IBD. We evaluated the ability of our model in discriminating IVCS-responding patients from non-responding patients with the area under the receiver operating characteristic curve (AUC) and the Youden index. Delong test compared the AUCs of our models with existing risk-scoring tools, the *P*-value adjustment was applied to control for the false discovery rate (FDR) based on the Benjamini–Hochberg procedure, and differences in validity between models were regarded as significant if the FDR-*P* < 0.05. We conducted the decision curve analysis (DCA) and the Hosmer–Lemeshow test for a comprehensive model evaluation. The built classifiers were internally validated using bootstrap 1000 replications to substantiate the generalization performance.

## Results

### Analysis of clinical characteristics

We incorporated 141 patients with confirmed severe IBD who received hospitalized IVCS. The response rate of IVCS was similar between the two subentities (59.42% for UC versus 65.28% for CD, *P* = 0.492). Compared to UC patients, patients with CD exhibited a younger age distribution (*P* < 0.001). Severe UC was associated with a higher incidence of CMV seropositivity than CD (*P* = 0.001). The UC population had a higher proportion of combined bloody stools (*P* < 0.001) as well as more frequent intestinal movements (*P* < 0.001) compared to CD patients. The parenteral condition was more prevalent in severe cases of CD than in UC (*P* = 0.002). Additionally, a greater degree of nutritional imbalance was observed in CD patients, as evidenced by lower body mass index and adiposity metrics. Table 1 summarizes differences in baseline characteristics between UC and CD.

**Table 1:** Comparisons of clinical information between UC and CD patients

Variables	Overall (*n* = 141)	Baseline characteristics		*P*
		**UC**	**CD**	
		**(*n* = 69)**	**(*n* = 72)**	
**Response, n (%)**	88(62.41%)	41(59.42%)	47(65.28%)	0.492
**Age (years)**	38.00(23.00–51.00)	48.00(33.50–55.50)	26.00(21.00–39.75)	< 0.001
**Male, n (%)**	85(60.28%)	38(55.07%)	47(65.28%)	0.233
**BMI (kg/m**^**2**^**)**	19.52 ± 3.46	20.54 ± 3.22	18.54 ± 3.43	0.001
**Temperature > 37.8 °C, n (%)**	45(31.91%)	19(27.54%)	26(36.11%)	0.285
**ESR (mm/h)**	50.23 ± 30.63	49.72 ± 30.80	50.71 ± 30.68	0.849
**CRP (mg/L)**	44.40(15.00–77.21)	55.10(14.99–85.78)	39.89(15.16–69.58)	0.338
**Hb (g/L)**	106.20 ± 25.79	107.93 ± 25.55	104.54 ± 26.08	0.438
**Alb (g/L)**	33.71 ± 6.92	33.37 ± 6.55	34.04 ± 7.29	0.566
**CMV seropositivity, n (%)**	10(7.09%)	10(14.49%)	0(0.00%)	0.001
**Disease duration (months)**	12.00(6.00–48.00)	12.00(4.00–57.00)	12.00(6.00–45.00)	0.831
**Stool frequency (n/day)**	6(4–10)	8(6–12)	5(2–8)	< 0.001
**Bloody stools, n (%)**	97(68.79%)	69(100.00%)	28(38.89%)	< 0.001
**Parenteral manifestations, n (%)**	42(29.79%)	12(17.39%)	30(41.67%)	0.002
**Colonic dilatation, n (%)**	15(10.64%)	11(15.94%)	4(5.56%)	0.057
**SATI (cm**^**2**^**/m**^**2**^**)**	24.36(12.44–39.87)	29.02(21.84–44.18)	19.84(9.18–33.94)	< 0.001
**VATI (cm**^**2**^**/m**^**2**^**)**	16.94(9.18–25.41)	18.66(12.27–31.60)	14.60(8.49–21.82)	0.027
**PSAI (cm**^**2**^**/m**^**2**^**)**	0.96(0.71–1.35)	1.19(0.96–1.57)	0.81(0.57–0.96)	< 0.001
**MATI (cm**^**2**^**/m**^**2**^**)**	4.33 ± 2.13	4.87 ± 2.08	3.81 ± 2.06	0.003
**MFI (cm**^**2**^**/m**^**2**^**)**	0.71(0.54–1.00)	0.68(0.56–0.81)	0.75(0.51–1.13)	0.090

Normally distributed continuous variables are described as means ± SDs, while non-normally distributed continuous variables are presented as medians with 25% and 75% quartiles. Categorized variables are given as absolute numbers and percentages

*UC* Ulcerative colitis, *CD* Crohn’s disease, *BMI* Body mass index, *ESR* Erythrocyte sedimentation rate, *CRP* C-reactive protein, *Hb* Hemoglobin, *Alb* Albumin, *CMV* Cytomegalovirus, *SATI* Subcutaneous adipose tissue index, *VATI* Visceral adipose tissue index, *PSAI* Paraspinal intramuscular adipose tissue index, *MATI* Mesorectal adipose tissue index, *MFI* Mesenteric fat index, *SDs* Standard deviations

Table 2 shows variations in clinical information between responders and non-responders. Traditional nutritional indicators, such as body mass index and albumin, decreased in steroid-refractory diseases. The disease location (*P*_UC_ = 0.321;* P*_CD_ = 0.544) and behavior (*P* = 0.239) were approximated among IVCS responders versus non-responders. UC and CD patients in more advanced clinical conditions tended to be steroid-insensitive (*P*_UC_ = 0.012;* P*_CD_ = 0.049). Among UC patients who responded well to IVCS, there was a notable increase in SATI, VATI, and MATI. Conversely, the cohort of CD patients effectively treated with IVCS showed diminished VATI and MFI. We further found that CD patients with a prolonged disease duration had a decreased SATI and an elevated MFI, while no correlation between disease process and fat parameters was observed in the UC population (Fig. S2).

**Table 2:** Baseline characteristics of the study population

Variables	Baseline characteristics of UC		*P*	Baseline characteristics of CD		*P*
	**Responders**	**Non-responders**		**Responders**	**Non-responders**	
	**(*n* = 41)**	**(*n* = 28)**		**(*n* = 47)**	**(*n* = 25)**	
**Age (years)**	50.00(32.50–55.50)	45.50(35.25–55.50)	0.956	25.00(22.00–41.00)	29.00(20.00–39.00)	0.696
**Male, n (%)**	20(48.78%)	18(64.29%)	0.228	29(61.70%)	18(72.00%)	0.444
**BMI (kg/m**^**2**^**)**	20.58(18.99–24.57)	19.35(17.41–21.54)	0.024	18.78 ± 2.83	18.09 ± 4.37	0.477
**Temperature > 37.8 °C, n (%)**	9(21.95%)	10(35.71%)	0.275	16(34.04%)	10(40.00%)	0.618
**ESR (mm/h)**	43.98 ± 30.00	58.14 ± 30.52	0.060	48.41 ± 30.62	55.05 ± 30.93	0.385
**CRP (mg/L)**	55.00(15.74–85.78)	55.86(14.65–90.91)	0.687	36.50(13.60–68.60)	48.60(19.58–74.46)	0.391
**Hb (g/L)**	102.98 ± 23.87	115.18 ± 26.62	0.051	105.98 ± 25.94	101.84 ± 26.67	0.525
**Alb (g/L)**	33.35 ± 6.49	33.40 ± 6.76	0.975	35.84 ± 6.64	30.66 ± 7.37	0.003
**CMV seropositivity, n (%)**	5(12.20%)	5(17.86)	0.729	0(0.00%)	0(0.00%)	-
**Disease duration (months)**	18.00(8.50–66.00)	12.00(1.25–24.00)	0.069	12.00(5.00–36.00)	16.00(6.50–48.00)	0.275
**Disease extent, n (%)**			0.321	-		
**Proctitis (E1)**	4(9.76%)	1(3.57%)				
**Left-sided colitis (E2)**	14(34.14%)	6(21.43%)				
**Extensive colitis (E3)**	23(56.10%)	21(75.00%)				
**Disease location, n (%)**	-					0.544
**Ileal (L1)**				9(19.15%)	3(12.00%)	
**Colonic (L2)**				13(27.66%)	5(20.00%)	
**Ileocolonic (L3)**				25(53.19%)	17(68.00%)	
**Disease behavior, n (%)**						0.239
**Inflammatory (B1)**				38(80.85%)	16(64.00%)	
**Stricturing (B2)**				6(12.77%)	7(28.00%)	
**Penetrating (B3)**				3(6.38%)	2(8.00%)	
**Stool frequency (n/day)**	7(6–12)	9(6–12)	0.319	5(3–7)	4(2–8)	0.392
**Bloody stools, n (%)**	41(100.00%)	28(100.00%)	-	20(42.55%)	8(32.00%)	0.452
**Parenteral manifestations, n (%)**	5(12.20%)	7(25.00%)	0.205	20(42.55%)	10(40.00%)	> 0.999
**Clinical severity (SCCAI/HBI)**	10(9–11)	11(10–12)	0.012	14(13–17)	16(16–18)	0.049
**Endoscopic severity (UECIS/SES-CD)**	6(6–7)	6(5–7)	0.290	16(14–20)	17(16–22)	0.184
**Colonic dilatation, n (%)**	4(9.76%)	7(25.00%)	0.106	2(4.26%)	2(8.00%)	0.606
**SATI (cm**^**2**^**/m**^**2**^**)**	42.29 ± 20.41	21.16 ± 9.64	< 0.001	16.21(9.92–32.65)	21.23(5.77–34.65)	0.882
**VATI (cm**^**2**^**/m**^**2**^**)**	24.43(13.46–41.53)	14.71(8.56–18.41)	0.001	11.63(6.50–17.89)	21.33(11.32–33.02)	0.004
**PSAI (cm**^**2**^**/m**^**2**^**)**	1.37(0.84–1.61)	1.15(0.98–1.34)	0.124	0.82(0.53–0.96)	0.79(0.65–1.05)	0.590
**MATI (cm**^**2**^**/m**^**2**^**)**	5.33 ± 1.79	4.20 ± 2.32	0.025	3.44(2.40–4.95)	3.14(1.69–6.27)	0.892
**MFI (cm**^**2**^**/m**^**2**^**)**	0.62(0.52–0.82)	0.73(0.61–0.82)	0.136	0.61(0.43–0.86)	1.11(0.88–1.49)	< 0.001

Normally distributed continuous variables are described as means ± SDs, while non-normally distributed continuous variables are presented as medians with 25% and 75% quartiles. Categorized variables are given as absolute numbers and percentages

*UC* Ulcerative colitis, *CD* Crohn’s disease, *BMI* Body mass index, *ESR* Erythrocyte sedimentation rate, *CRP* C-reactive protein, *Hb* Hemoglobin, *Alb* Albumin, *CMV* Cytomegalovirus, *SCCAI* Simple Clinical Colitis Activity Index, *HBI* Harvey-Bradshaw Index, *UCEIS* Ulcerative Colitis Endoscopic Index of Severity, *SES-CD* Simple Endoscopic Score for Crohn’s Disease, *SATI* Subcutaneous adipose tissue index, *VATI* Visceral adipose tissue index, *PSAI* Paraspinal intramuscular adipose tissue index, *MATI* Mesorectal adipose tissue index, *MFI* Mesenteric fat index, *SDs* Standard deviations

### The connection between fat indexes and disease activity

**Fig. 3:**
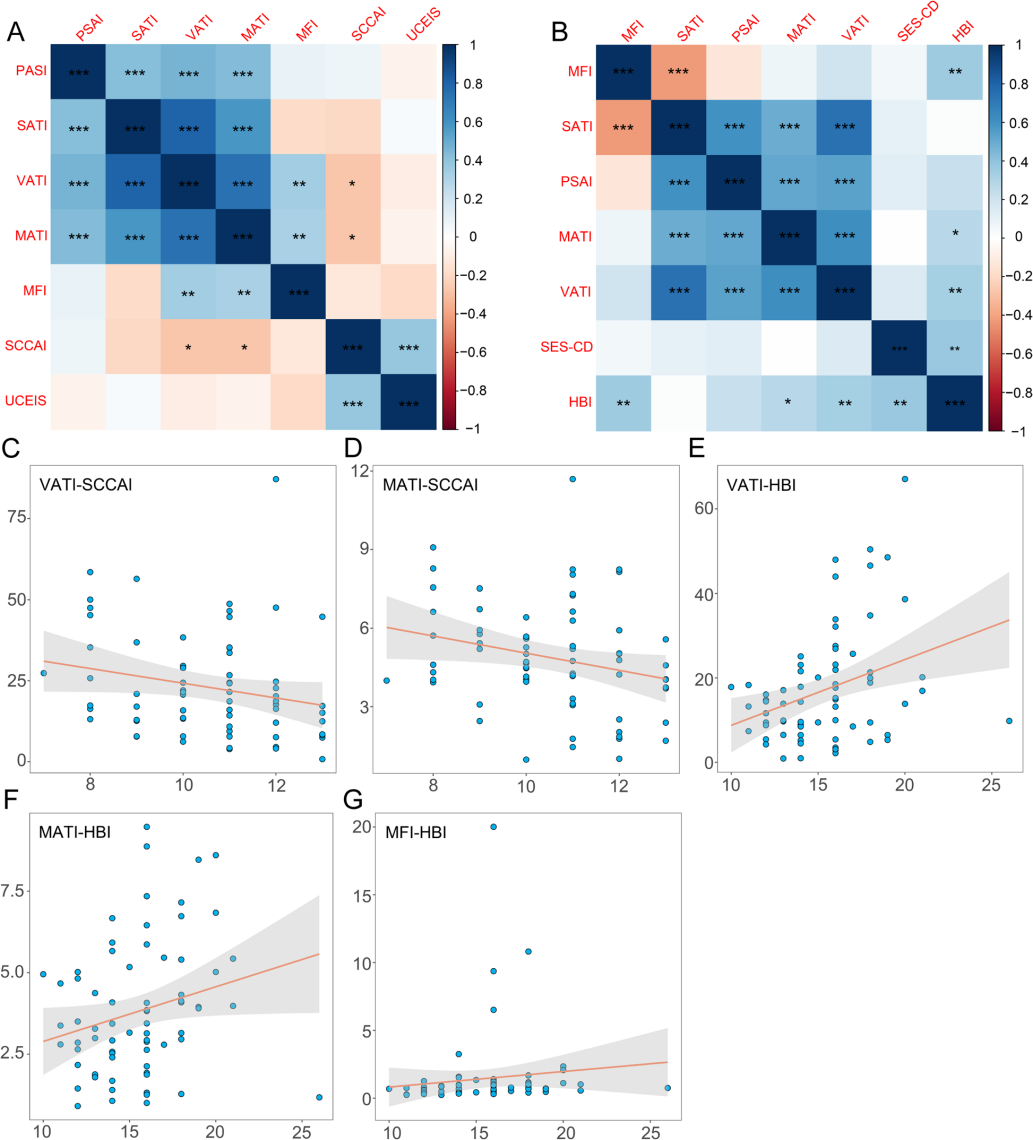
The correlation between fat metrics and disease severity. **A**, **B**. Correlations were produced using Spearman analysis. Positive correlations are expressed in blue, while orange reflects negative correlations. The graduated color shows the degree of correlation. * indicates *P* < 0.05, ** indicates *P* < 0.01, *** indicates *P* < 0.001. **C**–**G**. Fat parameters changed as UC and CD progressed. UC, ulcerative colitis; CD, Crohn's disease; SCCAI, Simple Clinical Colitis Activity Index; HBI, Harvey-Bradshaw Index; UCEIS, Ulcerative Colitis Endoscopic Index of Severity; SES-CD, Simple Endoscopic Score for Crohn's Disease; SATI, subcutaneous adipose tissue index; VATI, visceral adipose tissue index; PSAI, paraspinal intramuscular adipose tissue index; MATI, mesorectal adipose tissue index; MFI, mesenteric fat index

Leveraging Spearman correlation analysis, we attempted to disclose if body fat distribution was associated with the disease activity of IBD (Fig. 3). The Spearman correlation matrix revealed that the clinical activity and the endoscopic score of the study population were significantly correlated (rho_UC_ = 0.389, *P*_UC_ = 0.001; rho_CD_ = 0.373, *P*_CD_ = 0.001). For both UC and CD patients, the areas of different fat compartments were positively correlated, while they were independent of patients' mucosal condition. We identified correlations between clinical severity and fat indexes, revealing opposite directions in the two subtypes of IBD. The diminished visceral and mesorectal adipose tissue appeared to be detected in UC individuals with a more advanced stage (rho_VATI_ =  − 0.271, *P*_VATI_ = 0.024; rho_MATI_ =  − 0.270, *P*_MATI_ = 0.025). As CD clinically deteriorated, there was a significant increase in fat indexes with a coefficient of 0.331 for VATI (*P* = 0.005), 0.273 for MATI (*P* = 0.021), and 0.360 for MFI (*P* = 0.002).

### Multivariable logistic regression models for identifying effective IVCS

The count of variables in the regression equations was controlled to equal the number of adverse events divided by 5 − 10 owing to the constrained sample size [32]. Logistic regression models were constructed using physical metrics (SATI, VATI, PSAI, MATI, MFI) and conventional predictors of IVCS-induced remission such as C-reactive protein [33], albumin [34], colonic dilatation [35], and endoscopic severity [33]. When exploring risk factors of corticosteroid-refractory condition in patients with UC, we found that the VIF statistic of 5.916 for VATI was higher than 5, suggesting that it was not fit into the multivariable model (Table S1).

**Fig. 4:**
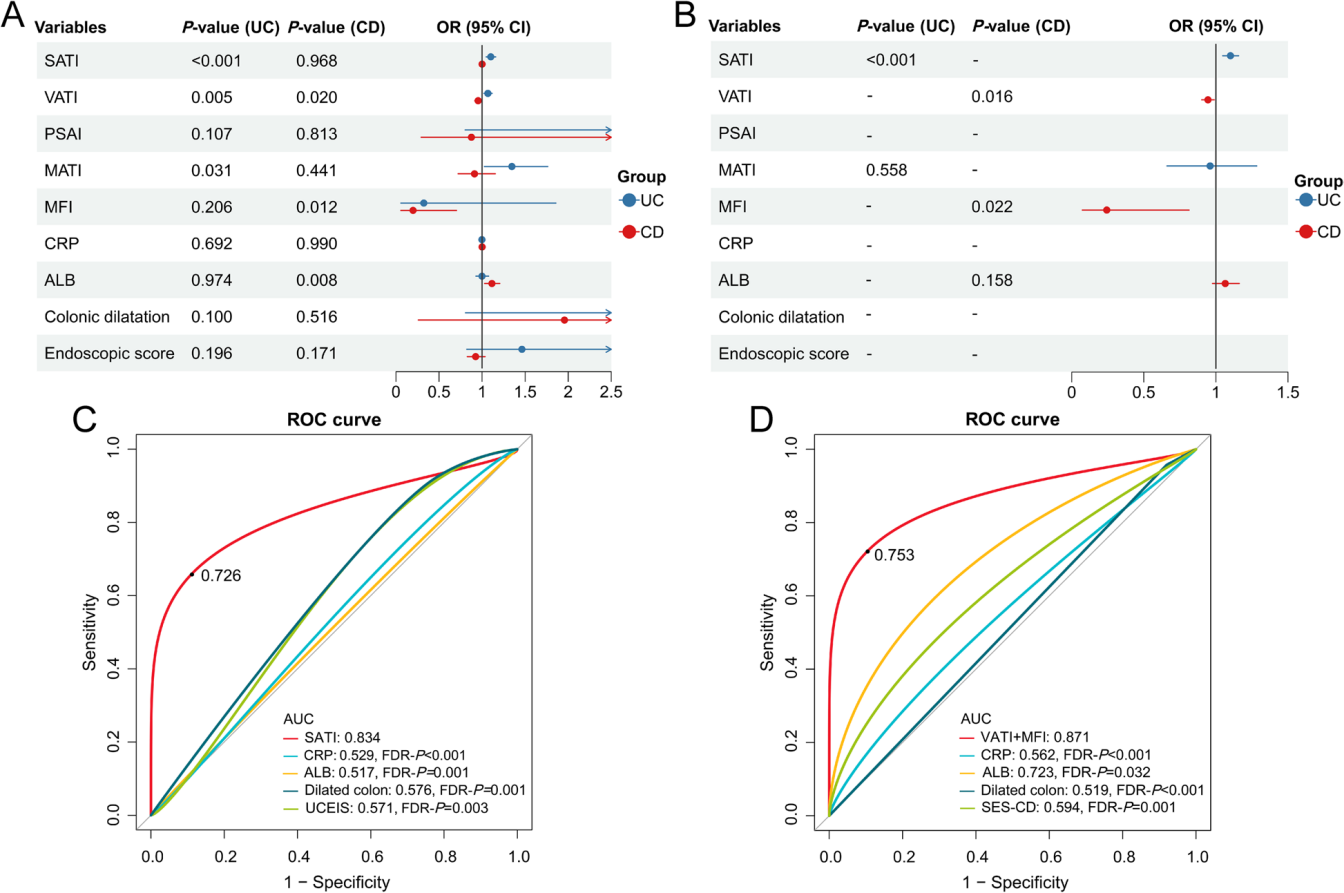
Identification of independent risk factors and assessment of the model’s prediction performance. **A**. The results of univariable logistic regression. The candidate risk factors for UC are SATI and MATI; for CD, the candidates are VATI, MFI, and ALB. **B**. The results of multivariable logistic regression. A high SATI index indicates that UC patients are more likely to be IVCS responders. For patients with CD, VATI and MFI are negatively associated with effective IVCS treatment. **C**, **D**. The built models were superior in identifying effective IVCS than traditional indicators. All FDR-*P* < 0.05. UC, ulcerative colitis; CD, Crohn’s disease; OR, odds ratio; CI, confidence interval; SATI, subcutaneous adipose tissue index; VATI, visceral adipose tissue index; PSAI, paraspinal intramuscular adipose tissue index; MATI, mesorectal adipose tissue index; MFI, mesenteric fat index; AUC, area under the receiver operating characteristic curve; CRP, C-reactive protein; ALB, albumin; UCEIS, Ulcerative Colitis Endoscopic Index of Severity; SES-CD, Simple Endoscopic Score for Crohn’s Disease; FDR, false discovery rate

After adjusting for confounders, we found that UC patients with a larger area of subcutaneous fat [odds ratio (OR): 1.101, 95% confidence interval (CI) 1.047–1.158; *P* < 0.001] had significantly higher odds of benefiting from IVCS (Fig. 4A and B). In the CD group, multivariable analysis revealed that as VATI (OR: 0.945, 95% CI 0.902 − 0.989; *P* = 0.016) and MFI (OR: 0.243, 95% CI 0.073 − 0.814; *P* = 0.022) increased, individuals were more susceptible to steroid-refractory conditions.

### Discriminative performance of fat indicators

We established optimal diagnostic cut-offs for SATI, VATI, and MFI in discriminating patients with IVCS-refractory IBD. Specifically, the threshold value of SATI was determined to be 36.099 cm^2^/m^2^ (Fig. S3A), while patients with VATI and MFI exceeding 18.417 cm^2^/m^2^ and 0.868 cm^2^/m^2^ were found to have an elevated propensity towards IVCS-insensitivity (Fig. S3B and C).

The multifactor classifiers composed of adiposity indexes demonstrated a satisfactory ability to identify IVCS-responding cases from the entire IBD population (AUC_UC_: 0.834, 95% CI 0.740–0.928; AUC_CD_: 0.871, 95% CI 0.793–0.949) (Fig. 4C and D). Resampling attempts yielded an AUC of 0.836 for UC (95% CI 0.735–0.919) and 0.876 for CD (95% CI 0.785–0.946), confirming the reliability and generalizability (Fig. S4). *P*-values of 0.208 and 0.093 derived from the Hosmer–Lemeshow test illustrated a strong consistency between the actual situations and the predicted values. The models provided net clinical benefits when the threshold probability ranged from 0.29 to 1.00 for UC and 0.31 to 0.95 for CD (Fig. S5). Baseline adiposity parameters exhibited superior performance (all FDR-*P*s < 0.05) in the early screening of corticosteroid-responding cases than existing risk-scoring tools (Fig. 4C and D).

## Discussion

The obesity epidemic has emerged as an unprecedented challenge throughout human society, fueling a growing interest in adipose tissue. The secretory property of adipose tissue underscores its significant role in the onset of autoimmune diseases, especially IBD. This study provides novel insights into the relationship between human fat indicators and IBD. Firstly, a notably different body fat distribution between UC and CD was observed. Additionally, adiposity distribution notably varied among populations who responded diversely to IVCS. The clinical disease activity of IBD was associated with fat components, and the correlation presented opposite trends in two subentities. Finally, our findings suggested that human adiposity metrics were beneficial in identifying steroid-refractory individuals, providing theoretical grounds for therapeutic regimens.

Considerable differences in baseline characteristics between UC and CD may be attributed to preferred anatomical locations of inflamed bowel. Characterized by discontinuous intestinal lesions, CD possibly involves the entire gastrointestinal system from the oral cavity to the anus. This condition particularly affects the small intestine, where nutrients are digested and absorbed [36]. As a result, patients with CD are more vulnerable to secondary nutritional deficits and fat depletion [37]. An increased frequency of bloody diarrhea in UC subjects is also related to a higher risk of rectal invasion. Intestinal CMV infection is a recognized trigger of steroid-refractoriness, and there should be histopathological evidence as the gold standard to prove its presence [38, 39]. As serum CMV replication cannot fully substitute for colonic CMV replication, the non-differential distribution of CMV seropositivity in the UC population can be explained.

The quantification of visceral fat at the third lumbar vertebra primarily encompasses retroperitoneal, omental, mesenteric, and mesocolic adipose tissue [40]. Accumulated mesenteric adiposity surrounding the inflamed intestine, also called creeping fat, proactively contributes to the pathogenesis of CD as a characteristic feature [41]. Proliferated mesenteric adiposity secretes numerous pro-inflammatory substances [12, 42], including interleukin‐1β, tumor necrosis factor-α, and interleukin‐6. These substances have long been acknowledged as stimuli of intestinal inflammation [42, 43]. However, creeping fat also exerts a protective and barrier influence due to overproduced anti-inflammatory cytokines and resident immune cells [43]. It is thus challenging to balance the role of creeping fat in the progression of CD. Our findings indicated that higher levels of VATI and MFI were associated with a more aggressive course of CD, suggesting that mesenteric fat will promote aberrant inflammation rather than suppress colitis, which is consistent with previously proposed hypotheses [14, 15]. Furthermore, we have observed a positive correlation between MATI and the clinical activity of CD, which represents a novel discovery. Mesorectal adipose tissue manifests as creeping fat deposition around the rectum [18]. Therefore, we hypothesize that expanded mesorectal adiposity leads to elevated levels of pro-inflammatory adipokines in local tissues, thereby aggravating proctitis.

In addition to potential inducers of severe CD, VATI and MFI were also positive imaging biomarkers of IVCS treatment failure. As the core modules of steroid-signaling pathways, structural and functional abnormalities of glucocorticoid receptors (GRs) are responsible for steroid insensitivity. First, when inflammatory agents are excessively generated, GRs may be nitrosylated, ubiquitinated, and phosphorylated, leading to instability, degradation, and malfunction [44]. Furthermore, pro-inflammatory adipokines activate transcription factors which in turn induce competition for cofactors or direct suppression of GRs through protein–protein interactions [45]. Additionally, elevated levels of inflammatory molecules stimulate the release of reactive oxygen species, which leads to oxidative modifications and impairments of GRs [45, 46]. Management of over-accumulated visceral and mesenteric fat (VATI > 18.417 cm^2^/m^2^; MFI > 0.868 cm^2^/m^2^) may be an alternative to enhance the therapeutic efficacy of IVCS.

Several small-scale studies have endeavored to elucidate the associations between adiposity metrics and disease phenotypes of UC. Some have recognized fat atrophy in patients with severe UC [13, 47], while Zhang *et* al. refuted this phenomenon [48]. In our analyses, both VATI and MATI exhibited a decreasing trend as colitis progressed. We also found that lower SATI was associated with an increased likelihood of requiring rescue therapies or surgeries, which contradicts a published observation [49]. Current studies, including our own, lack conclusive evidence on the effects of UC on fat metabolism due to the diverse ethnicities, ages, statures, disease severities, and durations within study populations. What is certain is that there is a nutritional imbalance in patients with severe UC. More aggravated systemic inflammation provokes absent IVCS response, excessive energy expenditure, overacted catabolism, and fatty dystrophy, while malnutrition inversely weakens the immune function of the gastrointestinal tract [50]. Supportive nutrition may break the vicious cycle between poor nutritional status and a consistently inflammatory environment. The potential of albumin and sarcopenia in diagnosing corticosteroid-refractory disease somewhat supports our hypotheses [34, 49].

Interestingly, the pro-inflammatory property of mesenteric fat appears to be attenuated in UC. CD-specific transmural lesions drive bacterial translocation, leading to hyperplasia and hypertrophy of mesenteric adipocytes, thereby achieving a peak of secretion and positive feedback in intestinal inflammation [43]. As UC typically damages the superficial colonic mucosal layer, subsequent physiological cascades are unlikely to occur.

The combinations of fat measures exhibited superior and robust performance in distinguishing individuals with varied medical responses, which were also clinically beneficial and highly aligned with actual situations. In our work, systemic steroids remained the first-line choice for those with a predicted probability of being an IVCS responder beyond 0.726 or 0.753.

### Study strengths and limitations

Increasing evidence states that adipose tissue plays a role in the development of IBD, with most research focused on CD. This study sheds light on changes in body fat composition as UC and CD progress, bridging gaps between nutritional imbalance and UC. Fat metrics accurately identify IBD patients who benefit from IVCS, potentially avoiding the adverse effects of ineffective ongoing treatment.

As an exploratory study, several deficiencies need to be confronted. First, the sample size was limited due to rigorous inclusion criteria, precluding subgroup analyses. Second, physical parameters were derived from CT slices, which might not account for individual variations even when multiple spinal levels were imaged. Furthermore, the study population consisted solely of Han Chinese patients with severe IBD, limiting generalizability. Our results will be more convincing if validated with large-scale prospective studies.

## Conclusions

In summary, CT-derived adiposity parameters were found to be correlated with the disease activity and reliably identified IBD patients who responded well to IVCS treatment. Therefore, human fat indexes are recommended as novel imaging biomarkers that can facilitate prompt rescue therapy regimens and prevent ineffective use of IVCS.

## Supplementary Information

**Additional file 1: Figure S1. **Consistency test of measurements. **Figure S2. **Correlation between disease duration and fat parameters. **Figure S3. **Diagnostic thresholds for predictors. **Figure S4. **Prediction accuracy derived from 1000 bootstrap resamplings. **Figure S5.** Clinical applicability of the established models. **Table S1. **Multicollinearity tests for candidate variables. 

## Data Availability

The clinical information is available after sending a reasonable request to the corresponding author.

## Abbreviations

IBD: Inflammatory bowel disease
UC: Ulcerative colitis
CD: Crohn’s disease
IVCS: Intravenous corticosteroids
CT: Computed tomography
SATI: Subcutaneous adipose tissue index
VATI: Visceral adipose tissue index
PSAI: Paraspinal intramuscular adipose tissue index
MATI: Mesorectal adipose tissue index
MFI: Mesenteric fat index
CMV: Cytomegalovirus
SCCAI: Simple Clinical Colitis Activity Index
HBI: Harvey-Bradshaw Index
UCEIS: Ulcerative Colitis Endoscopic Index of Severity
SES-CD: Simple Endoscopic Score for Crohn’s Disease
VIF: Variance inflation factor
AUC: Area under the receiver operating characteristic curve
FDR: False discovery rate
DCA: Decision curve analysis
OR: Odds ratio
CI: Confidence interval
GRs: Glucocorticoid receptors
