# Supplementary material for: Association between body fat composition and disease duration, clinical activity, and intravenous corticosteroid-induced response in inflammatory bowel disease
Source: Lipids Health Dis. 2023 Jul 22;22:106. doi: 10.1186/s12944-023-01874-4 (PMC10363311; doi:10.1186/s12944-023-01874-4)
Supplement: Supplementary file 1 — Additional file 1: Figure S1. Consistency test of measurements. Figure S2. Correlation between disease duration and fat parameters. Figure S3. Diagnostic thresholds for predictors. Figure S4. Prediction accuracy derived from 1000 bootstrap resamplings. Figure S5. Clinical applicability of the established models. Table S1. Multicollinearity tests for candidate variables. [file 12944_2023_1874_MOESM1_ESM.docx]

Supplementary Information

**Association between body fat composition and disease duration, clinical activity, and intravenous corticosteroid-induced response in inflammatory bowel disease**

Shubei He^1,2,3,4†^, Yuxia Huang^1,2,3,4†^, Ying Peng^1,2,3,4^, Jin Chai^1,2,3,4*^, Kun Chen^1,2*^

**
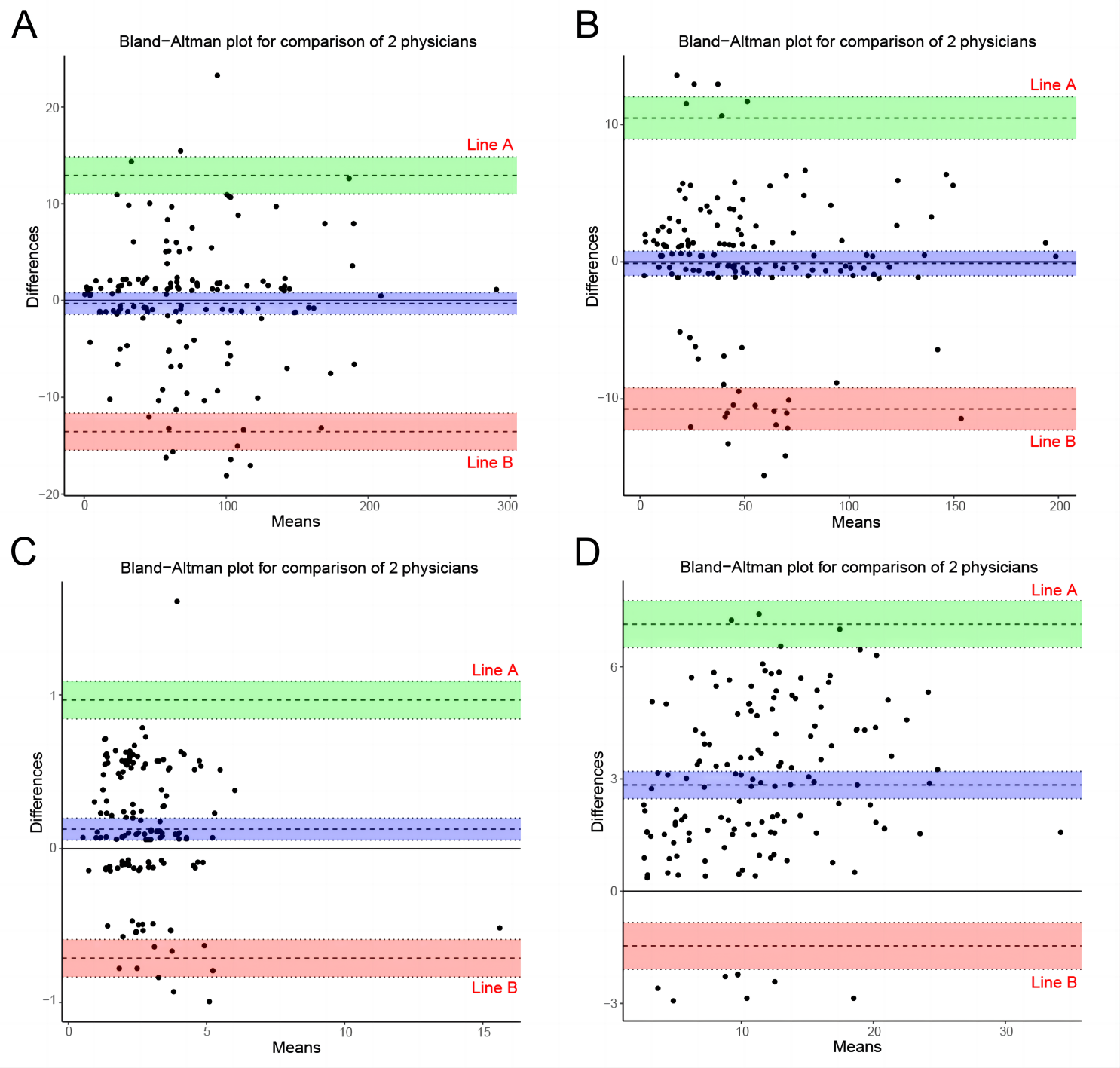
Figure S1.** Consistency test of measurements.

1. Measures of subcutaneous fat from two physicians. **(B)** Measures of visceral fat from two physicians. **(C)** Measures of paraspinal intramuscular fat from two physicians. **(D)** Measures of mesorectal fat from two physicians. Each black dot represents the difference between two physicians on the same indicator for the same patient. For all adipose metrics, 95% of the black dots are between Lines A and B, indicating a solid consistency.

**Figure S2.** Correlation between disease duration and fat parameters.

**
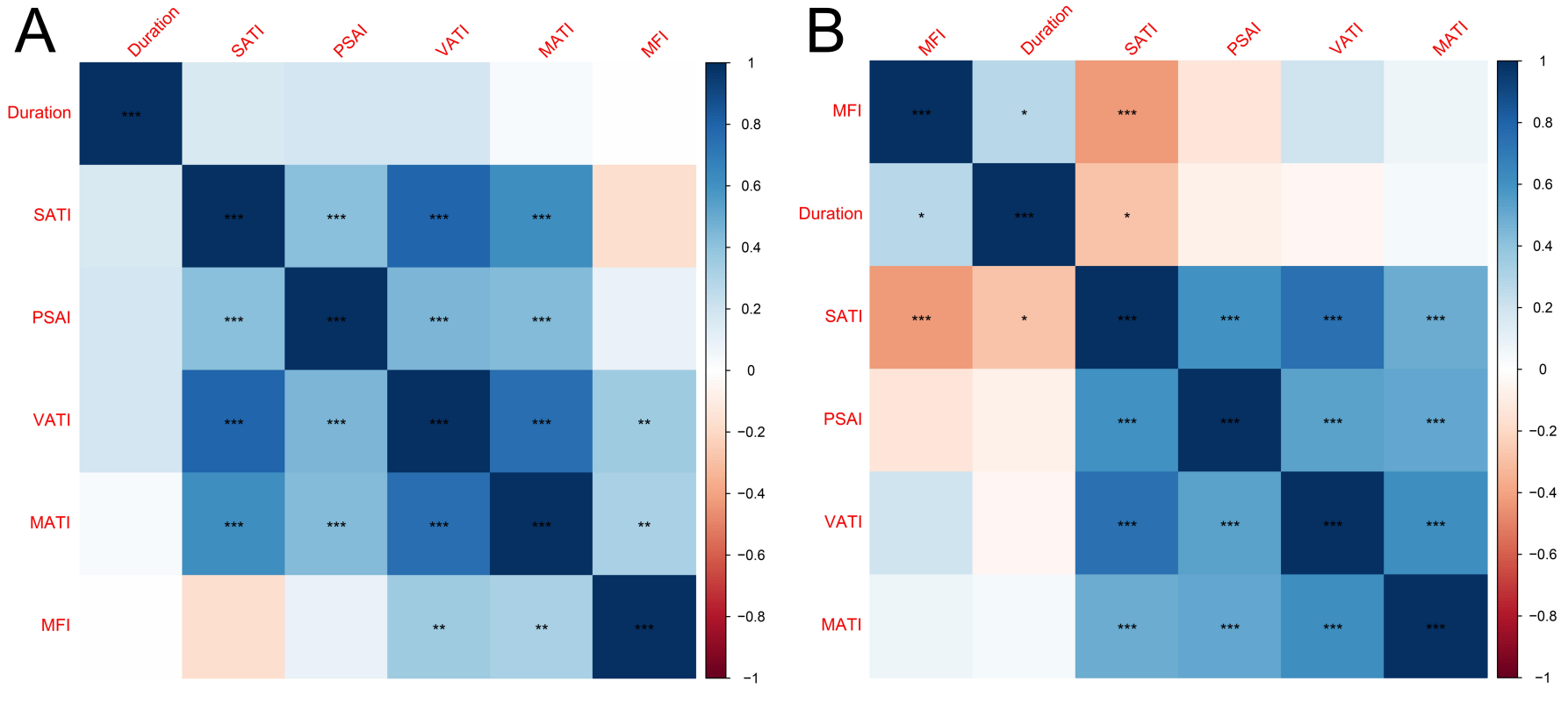
(A)** Spearman correlation analysis conducted in UC patients. **(B)** Spearman correlation analysis conducted in CD patients. According to the individual disease duration, patients were divided into group 1 (< 12 months), group 2 (12 to 24 months), and group 3 (> 24 months). UC, ulcerative colitis; CD, Crohn's disease. SATI, subcutaneous adipose tissue index; VATI, visceral adipose tissue index; PSAI, paraspinal intramuscular adipose tissue index; MATI, mesorectal adipose tissue index; MFI, mesenteric fat index.

**Table S1.** Multicollinearity tests for candidate variables.

| **Variables** | **Variance inflation factor (UC)** | **Variance inflation factor (CD)** |
| --- | --- | --- |
| **C-reactive protein** | 1.088 | 1.327 |
| **Albumin** | 1.098 | 1.570 |
| **Colonic dilatation** | 1.133 | 1.086 |
| **Endoscopic severity** | 1.162 | 1.058 |
| **SATI** | 4.437 | 3.028 |
| **VATI** | 5.916 | 3.172 |
| **PSAI** | 1.676 | 3.214 |
| **MATI** | 1.724 | 2.057 |
| **MFI** | 1.463 | 1.222 |

UC, ulcerative colitis; CD, Crohn's disease; SATI, subcutaneous adipose tissue index; VATI, visceral adipose tissue index; PSAI, paraspinal intramuscular adipose tissue index; MATI, mesorectal adipose tissue index; MFI, mesenteric fat index.

**
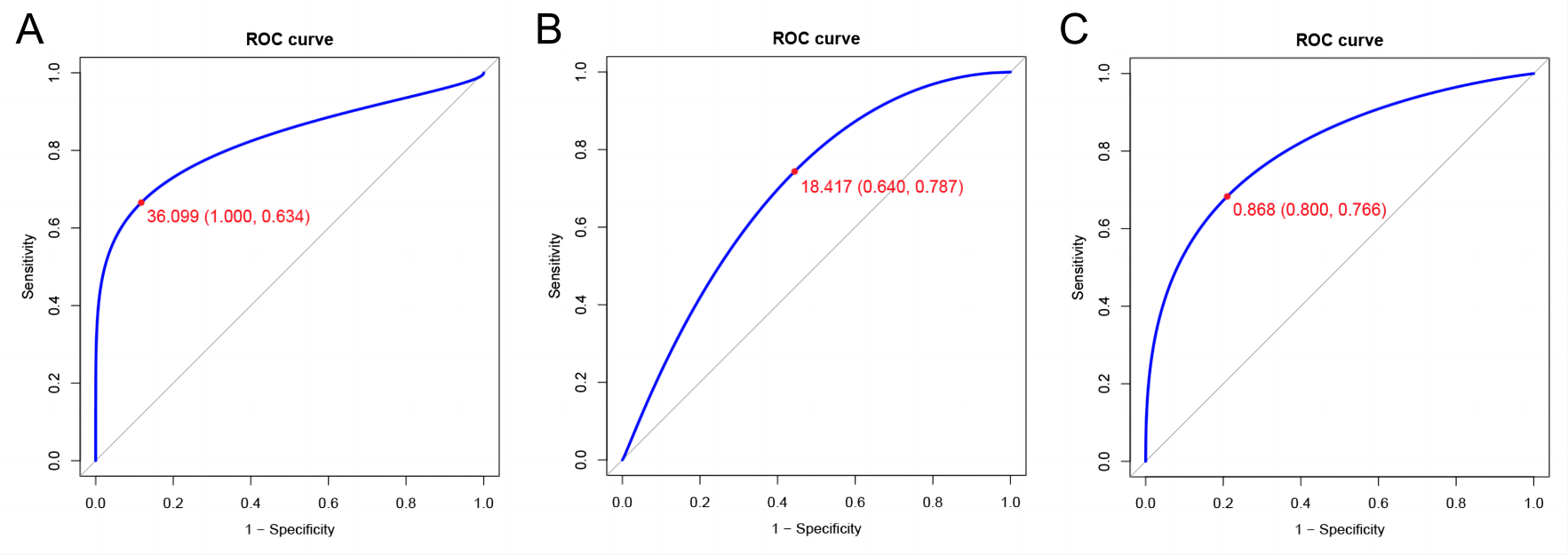
Figure S3.** Diagnostic thresholds for predictors.

1. The diagnostic threshold of SATI was 36.099 cm^2^/m^2^, demonstrating optimal sensitivity of 0.634 and specificity of 1.000. **(B)** The diagnostic threshold of VATI was 18.417 cm^2^/m^2^, demonstrating optimal sensitivity of 0.787 and specificity of 0.640. **(C)** The diagnostic threshold of MFI was 0.868 cm^2^/m^2^, demonstrating optimal sensitivity of 0.766 and specificity of 0.800. ROC, receiver operating characteristic curve; SATI, subcutaneous adipose tissue index; VATI, visceral adipose tissue index; MFI, mesenteric fat index.

**
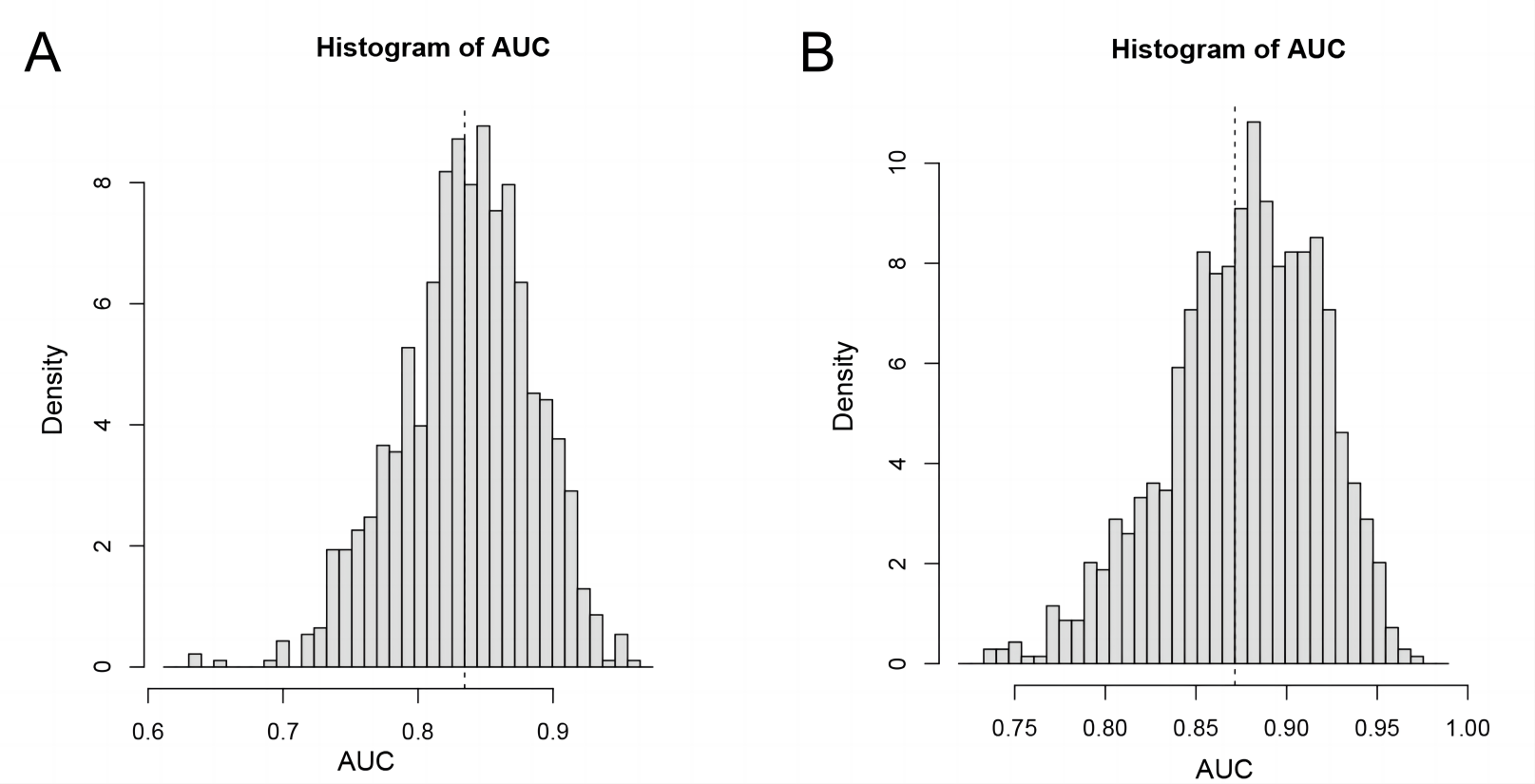
Figure S4.** Prediction accuracy derived from 1000 bootstrap resamplings.

**(A)** Distribution of 1000 resampled AUCs for UC. **(B)** Distribution of 1000 resampled AUCs for CD. In the histograms, the dotted vertical line indicates the median AUCs. The mean AUCs were 0.836 for UC and 0.876 for CD, respectively. AUC, area under receiver operating characteristic curve; UC, ulcerative colitis; CD, Crohn's disease.

**
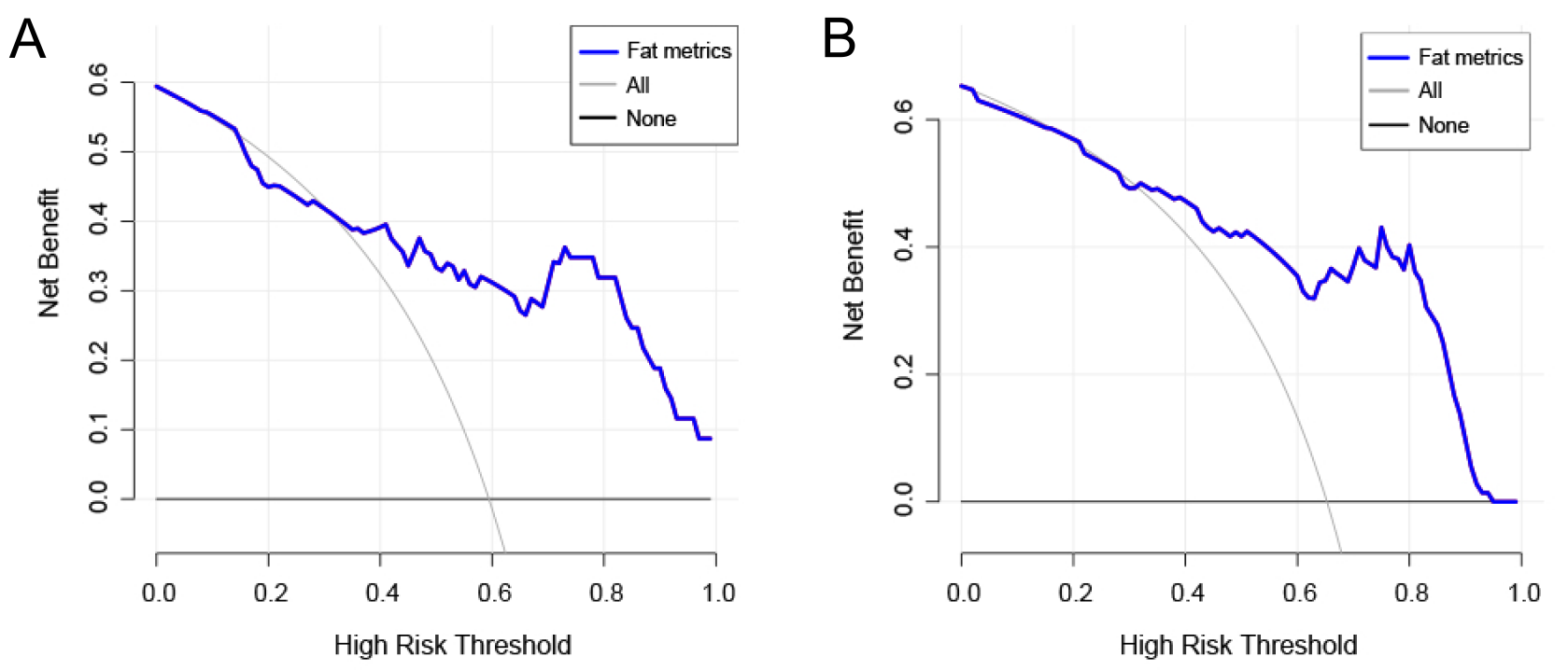
Figure S5.** Clinical applicability of the established models.

**(A)** The DCA plot of UC. **(B)** The DCA plot of CD. In the DCA plots, good clinical applicability appears when the blue line is further from the All and None lines. DCA, decision curve analysis; UC, ulcerative colitis; CD, Crohn's disease.
